# Exploring the molecular interface between hypoxia-inducible factor signalling and mitochondria

**DOI:** 10.1007/s00018-019-03039-y

**Published:** 2019-02-14

**Authors:** Luke W. Thomas, Margaret Ashcroft

**Affiliations:** University of Cambridge, Cambridge Biomedical Campus, Cambridge, CB2 0AH UK

**Keywords:** Hypoxia, HIF, Metabolism, Oxygen, Oxphos, Mitochondrial biogenesis, Respiratory chain

## Abstract

Oxygen is required for the survival of the majority of eukaryotic organisms, as it is important for many cellular processes. Eukaryotic cells utilize oxygen for the production of biochemical energy in the form of adenosine triphosphate (ATP) generated from the catabolism of carbon-rich fuels such as glucose, lipids and glutamine. The intracellular sites of oxygen consumption-coupled ATP production are the mitochondria, double-membraned organelles that provide a dynamic and multifaceted role in cell signalling and metabolism. Highly evolutionarily conserved molecular mechanisms exist to sense and respond to changes in cellular oxygen levels. The primary transcriptional regulators of the response to decreased oxygen levels (hypoxia) are the hypoxia-inducible factors (HIFs), which play important roles in both physiological and pathophysiological contexts. In this review we explore the relationship between HIF-regulated signalling pathways and the mitochondria, including the regulation of mitochondrial metabolism, biogenesis and distribution.

## Introduction

In humans (and other higher eukaryotes) the availability of molecular oxygen is an important determinant of biological outcomes in both physiological and pathophysiological processes, ranging from vascular patterning and tissue architecture during development, to the proliferation, invasiveness and metastasis of malignant cells in cancer. Oxygen is important for many cellular processes by cells to produce biochemical energy in the form of adenosine triphosphate (ATP) [1]. The sites of oxygen consumption-coupled ATP production in the cell are the mitochondria, which are therefore centrally important organelles for cell survival, as well as in influencing oxygen availability both inside and outside the cell. There are numerous non-mitochondrial processes which also generate ATP, such as glycolysis [1], and the enzyme creatine kinase (CK) which plays an important role in buffering ATP levels in tissues with high energetic demands such as skeletal muscle [2, 3]. Furthermore, a small number of eukaryotes are facultative anaerobes, and can generate ATP and survive in environments with little or no oxygen, such as yeast [4] and aquatic invertebrates [5].

In humans, reductions in the availability of oxygen (hypoxia) are sensed directly and indirectly by several cellular signalling pathways, which elicit a variety of transcriptional, metabolic and morphological responses to maintain cellular homeostasis. The major transcriptional regulators of the response to hypoxia consist of a highly evolutionarily conserved oxygen-regulated family of transcription factors, named the hypoxia-inducible factors (HIFs) [6, 7]. The HIFs are dimeric transcription factors that consist of a HIF-α subunit (HIF-1α or HIF-2α) which is rapidly degraded in an oxygen-dependent manner [6, 8–10], and a proteolytically stable beta subunit [7, 11]. The alpha subunit is continuously synthesised in the cytosol, where it is rapidly degraded by the 26S proteasome under normoxic conditions [10]. When oxygen is limiting, HIF-α is stabilised, and translocates to the nucleus, where it binds to conserved sequences (RCGTG) in the promoter regions of HIF-regulated genes [9, 12, 13], which are named hypoxia response elements (HREs). Transcriptional transactivation of genes is dependent on the association of the HIF-α with the HIF-1β subunit, also known as ARNT (aryl-hydrocarbon-receptor nuclear translocator) [14, 15], as well as other coactivators such as CBP/p300 (CREB-binding protein/adenovirus E1A-binding protein p300) [16].

The transcriptional targets of HIF include genes involved in cell survival and metabolism [17], and are thus essential for the adaptation of cells to hypoxia. A growing number of HIF targets directly or indirectly influence mitochondrial biology, and there is a reciprocal relationship between mitochondria and the HIF pathway. In this review, we will explore the relationship between HIF and mitochondria, with particular focus on cancer. It is important to note, however, that HIFs have significant cell-specific roles in non-transformed cells, resulting in different outcomes under hypoxic stress, e.g. the hypoxic preconditioning of cardiomyocytes and neurons in ischaemic disease [18, 19], the metabolic adaptation of skeletal muscle to altitude-related hypoxia [20], and the enhancement of neutrophil lifespan in hypoxic niches [21].

## Mitochondria, oxygen consumption and energy production

**Fig. 1:**
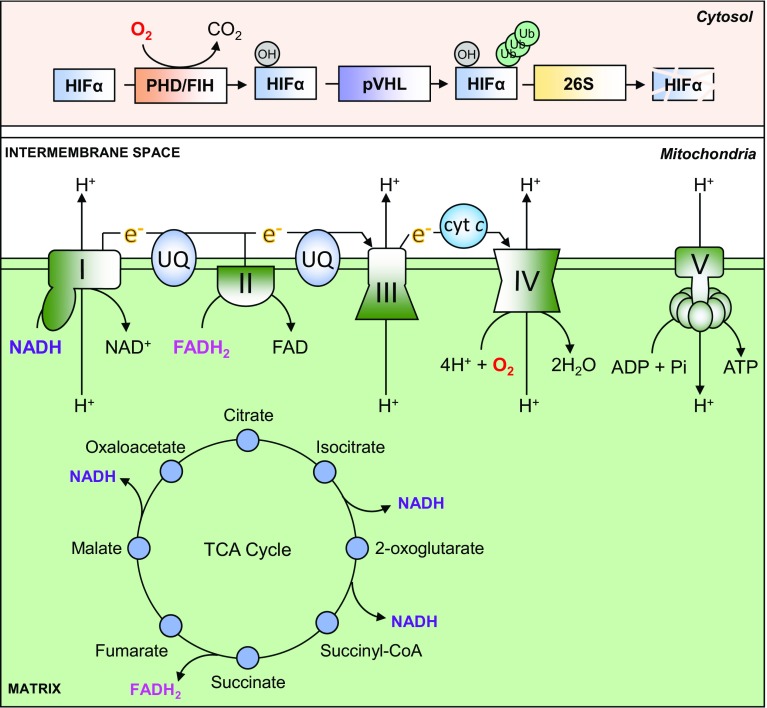
Oxygen-dependent ATP synthesis and HIF-α degradation. ATP is synthesised in the mitochondria by an *F*_1_*F*_0_-type ATP synthase, also known as CV (V). It uses energy provided by an electrochemical gradient formed through proton (H +) pumping between the mitochondrial matrix and the intermembrane space, which is carried out by CI, CIII and CIV. Proton transfer against this electrochemical gradient is energetically unfavourable, and is therefore powered by serial transfer of electrons (e–) from CI and CII to CIII via ubiquinone (UQ), and from CIII to CIV via cytochrome *c* (cyt *c*). CIV combines these electrons with molecular oxygen (O2) and protons to produce water (H2O). Electrons are provided to the ETC by the reducing equivalents NADH (to CI) and FADH2 (to CII), which are produced at various steps in the TCA cycle. Oxygen is also used to regulate the stability of HIF-α subunits. In the presence of oxygen, PHD and FIH enzymes hydroxylate specific residues on HIF-α proteins, which permit their recognition and ubiquitination by pVHL. Polyubiquitination of HIF-α proteins then targets them for degradation by the 26S proteasome

The eukaryotic mitochondrion is hypothesised to have arisen as the result of an endosymbiotic fusion between an archaeal host cell and a protobacterium that had evolved the ability to generate chemical energy through oxidative phosphorylation (OXPHOS) [22, 23]. One fundamental outcome of mitochondrial endosymbiosis was the acquisition by eukaryotes of the means to harness the oxidative power of molecular oxygen to efficiently generate large quantities of energy in the form of ATP, through enzymatic means. The mitochondrial ATP synthase is an *F*_1_*F*_0_-type ATP synthase, which catalyses the phosphorylation of adenosine diphosphate (ADP) to ATP, is powered by a proton-motive gradient between the inner matrix of the mitochondria and the inter-membrane space (IMS). This proton gradient is formed by the action of three protein complexes, namely Complex (C)I, CIII and CIV, which ‘pump’ protons against the gradient from the matrix into the IMS. The proton-motive action of CI, CIII and CIV is energetically unfavourable, and therefore requires energy in the form of serial transmission of electrons between the complexes through two intermediaries, ubiquinone (CI and CII–CIII) and cytochrome *c* (CIII–CIV), which together is referred to as the electron transport chain (ETC), or respiratory chain. Electrons are supplied to the ETC by a sequence of reactions in the matrix of the mitochondria termed the tricarboxylic acid (TCA) cycle, which produces three reducing (electron donating) equivalents of NADH, and one reducing equivalent of FADH_2_. The terminal electron acceptor in the chain is CIV, which combines molecular oxygen, protons and the electrons received from CIII via cytochrome *c*, to produce water. Energy can also be produced through the glycolytic metabolism of glucose to pyruvate in the absence of oxygen, and pyruvate can then undergo anaerobic fermentation to lactate, rather than undergoing oxidation in the mitochondria. However, the yield of ATP from glycolysis alone is only 2 molecules per molecule of glucose consumed, compared with 30–38 molecules of ATP through glycolysis combined with oxidative phosphorylation [1]. Thus, oxidative phosphorylation maximises the release of energy stored in carbon-rich fuels such as glucose for use by the cell (Fig. 1).

As the major oxygen-consuming organelle of the cell, mitochondria are uniquely dependent on the continued availability of oxygen for ETC function. Indeed, it has been estimated that as much as 90% of cellular oxygen consumption is carried out by cytochrome *c* oxidase (CIV), which has a very high affinity for oxygen, with a *K*_m_ in the sub-micromolar range [24]. As a consequence, adaptations to hypoxia have evolved for cells to maintain bioenergetic homeostasis, while minimising the harmful effects of decreased oxygenation such as reactive oxygen species (ROS) production. Many of these adaptations involve communications to and from mitochondrial metabolic pathways as well as changes in mitochondrial morphology, dynamics and subcellular localization. Adaptations to hypoxia are exquisitely controlled by HIF and the hypoxia-response machinery of the cell, which includes various signalling pathways and gene expression changes regulated by HIF.

## Oxygen-dependent regulation of HIF signalling

The transcriptional activity of HIF-α proteins is regulated in an oxygen-dependent manner by controlling their protein stability in the cytosol, and through regulation of their binding to transcriptional coactivators in the nucleus. Regulation at both levels is mediated by the hydroxylation of specific amino acids by oxygen-dependent dioxygenases. Two classes of dioxygenases are capable of hydroxylating HIF-α proteins: the proline-targeting prolyl hydroxylase domain (PHD)-containing enzymes (1, 2 and 3) [25–27], and the asparagine-targeting factor inhibiting HIF (FIH) enzyme [28–30]. Both classes of enzyme catalyse the oxidative decarboxylation of 2-oxoglutarate [2-OG, or α-ketoglutarate (α-KG)], which produces carbon dioxide, and succinate as by-products. Iron in the ferrous (Fe^2+^) oxidation state is also required, which is maintained in this state by the reducing action of ascorbic acid [31, 32]. Proline hydroxylation of HIF-α subunits by the PHDs permits their recognition and binding by the von-Hippel Lindau protein (pVHL) [10, 25, 26] (also see Fig. 1), which is the recognition component of a multimeric ubiquitin ligase. pVHL, along with elongin B (TCEB2), elongin C (TCEB1), cullin 2 (CUL2) and ring-box 1 (RBX1), is responsible for the ubiquitination of HIF-α subunits which targets the protein for proteasomal degradation [33]. Loss of pVHL activity through mutation leads to constitutive stabilisation of HIF-α proteins in normoxia, which contributes to disease progression in the tumour syndrome von-Hippel Lindau Disease [34, 35]. Hydroxylation by FIH does not affect HIF-αprotein stability, but rather inhibits the interaction between HIF-α subunits and the transcriptional co-activator, CREB-binding protein (CBP/p300) [28–30], which inhibits the transactivation of target genes by HIFs. In conditions of hypoxia, the hydroxylase activity of the PHDs and FIH is inhibited, which blocks the binding and ubiquitination of HIF-α proteins by pVHL, leading to cytoplasmic stabilisation of the HIF-α subunits [25–29]. Accumulated HIF-α translocates to the nucleus, where they then bind to HREs, dimerise with HIF-β subunits and recruit additional transcriptional co-activators to transactivate the transcription of hypoxia-responsive genes [36, 37].

## HIF-dependent regulation of mitochondria

The reduction in oxygen availability under hypoxia means that cells must adapt their metabolic programme to maintain the catabolic and anabolic reactions that rely on the availability of ATP normally supplied by OXPHOS. In general, HIF-1 signalling is considered to support anaerobic ATP production and downregulate OXPHOS, thus reducing the cell’s reliance on oxygen-dependent energy production [38]. Indeed, this metabolic reprogramming under hypoxia was one of the first functions ascribed to HIF-1 activity [8, 39, 40]. While there is evidence that HIF-1α and HIF-2α have some opposing roles when co-expressed, with relation to mitochondrial function, both have been shown to act to decrease a cell’s dependence on mitochondrial OXPHOS in a similar manner [41]. For example, in the absence of HIF-1α, as in the case of certain renal cell carcinomas, HIF-2α instigates the same remodelling of cellular metabolism away from OXPHOS and towards anaerobic means of ATP production [42, 43].

### *Suppression of the TCA cycle and ETC activity*

**Fig. 2:**
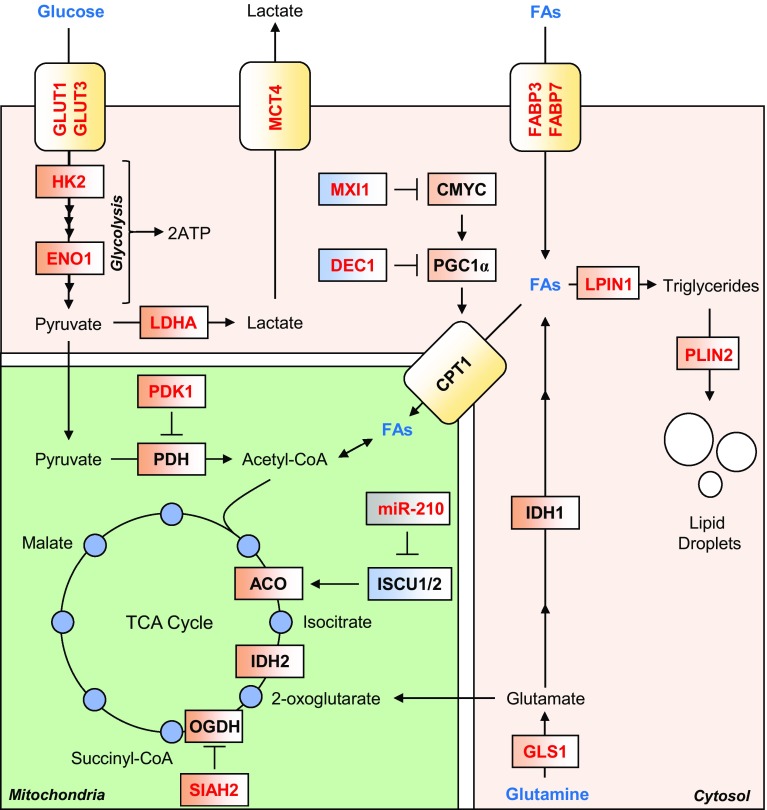
HIF-mediated changes to mitochondrial carbon metabolism. HIF signalling mediates an increase in anaerobic ATP production, by increasing glycolysis rates, through increased expression of glucose transporters GLUT1 and GLUT3, and almost all glycolytic enzymes, such as HK2 and ENO1. HIF signalling also diverts glucose-derived pyruvate away from mitochondrial respiration by increasing expression of LDHA, and PDK1, a negative regulator of PDH. Lactate efflux is increased by HIF-mediated increase in expression of MCT4. Fatty acids (FAs) are also diverted from catabolism to acetyl-CoA, through suppression of the rate-limiting enzyme in the mitochondrial import of FAs, CPT1. This is achieved through HIF-dependent upregulation of two negative regulators of PGC-1α expression, namely MXI1 and DEC1. FA import into the cell is increased by HIF-dependent upregulation of FABP3 and FABP7, and their conversion to triglycerides and lipid droplets is increased, through upregulation of LPIN1 and PLIN2, respectively. Glutamine is diverted from oxidation by the TCA cycle, through degradation of the 2-OG metabolising enzyme OGDH, by HIF-mediated increase in the expression of the OGDH-targeting SIAH2. This increases 2-OG availability for reductive carboxylation via IDH 1 and IDH2, which produces lipogenic acetyl-CoA. Glutamine flux to acetyl-CoA is also increased by HIF-dependent upregulation of GLS1. Conversely, oxidation of acetyl-CoA to 2-OG is suppressed through HIF-dependent upregulation of mir-210, which downregulates ISCU1/2, which is required for the activity of ACO

As mentioned above, the TCA cycle is a series of enzymatically catalysed reactions in the mitochondrial matrix, that provide electrons to the ETC, in the form of the reducing equivalents NADH and FADH_2_. TCA cycle intermediates are derived from external carbon sources, whose catabolism provides transitional metabolites that enter the TCA cycle at different points. Three major metabolites are used to provide carbons to replenish TCA cycle intermediates, namely glucose and fatty acids which are catabolised to acetyl-CoA, and glutamine, which in part is catabolised to succinyl-CoA via 2-OG by the TCA cycle. In hypoxia, HIF-regulated gene expression diverts glucose and fatty acid-derived carbons from being catabolised to acetyl-CoA, while glutamine-derived carbons are diverted from being catabolised to succinyl-CoA (Fig. 2).

To maintain ATP production from glycolysis in the context of decreased oxidative phosphorylation, HIF upregulates lactate dehydrogenase (LDHA) [8], an enzyme responsible for the conversion of pyruvate to lactate, in a reaction which regenerates NAD (Fig. 2). This diverts pyruvate away from conversion to acetyl-CoA in the mitochondria and the TCA cycle, and suppresses ETC activity. HIF also upregulates pyruvate dehydrogenase kinase 1 (PDK1), an enzyme responsible for the phosphorylation and inactivation of the mitochondrial enzyme pyruvate dehydrogenase (PDH) [38, 44]. PDH is responsible for the mitochondrial conversion of pyruvate to acetyl-CoA, and without PDH activity more pyruvate is available for conversion to lactate by LDHA. In addition, HIF upregulates monocarboxylate transport 4 (MCT4), a plasma membrane transporter which exports the increased levels of lactate out of the cell to maintain optimal cytoplasmic pH [45], and avoid competitive inhibition of LDHA. Lactate produced under hypoxic conditions is not merely a waste product, and can be re-oxidised by cells to pyruvate via LDHA, and thus contribute towards gluconeogenesis or oxidative phosphorylation upon reoxygenation [46–48]. In brain tissues, there is evidence to suggest the existence of a lactate shuttle whereby lactate exported from astrocytes under hypoxia can then be imported and utilised as a fuel by neurons [49, 50]. Similarly, lactate produced by skeletal muscle can be used as a fuel source by heart muscle under exercise-induced hypoxia [51]. Thus, HIF-mediated upregulation of LDHA under hypoxia can, in certain tissues, both decrease and increase mitochondrial activity to support energetic homeostasis. HIF also upregulates plasma membrane glucose transporters (GLUT1, GLUT3) [40, 52, 53] and glycolytic enzymes such as hexokinase 2 (HK2) [54], aldolase A (ALDA) and enolase 1 (ENO1) [8]) to increase glycolytic flux and thus maintain ATP homeostasis, since glycolytic ATP production is less efficient (2 ATP/glucose molecule) than OXPHOS (30-36 ATP/glucose molecule).

Alongside glucose, lipids can be catabolised to produce acetyl-coA to replenish the TCA in a process called β-oxidation, which occurs almost exclusively in the mitochondria [55]. Indeed, when rates of glycolysis are decreased, β-oxidation is upregulated to ensure a continued supply of acetyl-CoA to support OXPHOS [56]. However, in the absence of oxygen, this process is inhibited by reduced respiratory chain activity, and through HIF-mediated suppression of peroxisome proliferator-activated receptor gamma coactivator 1 alpha (PGC-1α), a transcriptional co-activator and critical regulator of lipid homeostasis [57] (described in more detail below) (Fig. 2). One of the transcriptional targets of PGC-1α is the mitochondrial protein carnitine palmitoyltransferase I (CPT1) [58] which catalyses the rate-limiting step in oxidation of long-chain fatty acids (C8 +), required for their mitochondrial import. Depletion of HIF-1α or HIF-2α in cultured hepatic cells has been shown to block the hypoxic suppression of PGC-1α-regulated gene expression, including genes involved in lipid catabolism [59] (such as CPT1), while HIF-2α (*Epas1*) deletion, but not HIF-1α deletion, was found to have the same effect in hepatic mouse tissue [60].

HIF activity therefore suppresses the synthesis of both glucose-derived and fatty acid-derived acetyl-CoA through multiple means. In addition, HIF-1 has been shown to regulate the expression of proteins involved in the import of extracellular fatty acids across the plasma membrane, such as fatty acid-binding protein 3 and 7 (FABP3, FABP7) [61, 62], as well as enzymes involved in lipid storage, such as perlipin 2 (PLIN2) [63]. HIF-1 also regulates the expression of lipin-1 (LPIN1), an enzyme which catalyses the penultimate step in triglyceride synthesis [64]. As a consequence of decreased mitochondrial lipid catabolism and elevated lipid import, synthesis and storage, lipid accumulation in droplets is commonly observed in multiple cell types under hypoxia [65–67] (Fig. 2). Hypoxic lipid accumulation appears to be a precautionary survival mechanism in cancer cells, to protect from ROS-mediated damage and cell death during reoxygenation [63].

A further way that HIFs support lipid synthesis is through the stimulation of glutamine catabolism to replenish TCA cycle intermediates, and ultimately generate lipogenic acetyl-CoA. Two distinct pathways exist to generate acetyl-CoA from glutamine, both the canonical conversion of glutamine-derived 2-OG to acetyl-CoA via malate [68], as well as the reductive carboxylation of 2-OG to produce acetyl-CoA via citrate through the reductive reverse reaction of isocitrate dehydrogenase (IDH) [69]. Under hypoxia, the canonical oxidative route is inhibited by HIF through the upregulation of siah E3 ubiquitin ligase 2 (SIAH2), a mitochondrial ubiquitin ligase which leads to the proteolytic destruction of the TCA cycle enzyme oxoglutarate dehydrogenase (OGDH) [70]. OGDH catalyses the conversion of 2-OG to succinyl-CoA, and thus decreased OGDH activity increases the concentration of 2-OG derived from glutamine (Fig. 2). The accumulated 2-OG can then be converted back to acetyl-CoA through reductive carboxylation catalysed by the isocitrate dehydrogenase (IDH) enzymes, IDH1 (cytosolic) and IDH2 (mitochondrial). Indeed, OGDH knock-out leads to normoxic stabilisation of HIF-α proteins, highlighting the importance of this enzyme in the relationship between HIF and mitochondria [71]. Furthermore, HIF upregulates the enzyme glutaminase 1 (GLS1) [72], which is responsible for the conversion of glutamine to glutamate, thus increasing the flux of glutamine to 2-OG, and on to lipogenic acetyl-CoA. While the precise contribution of HIF activity to glutamine-dependent lipid synthesis is unclear, both constitutive HIF-1 and HIF-2 signalling appear to be able to stimulate this metabolic shift [73].

### *Hypoxic upregulation of microRNAs*

A number of microRNAs (miRNAs) have been identified as regulators of genes involved in mitochondrial function, morphology and biogenesis (reviewed in [74]). One such miRNA is the HIF-upregulated miR-210 [75], which is commonly considered as the major hypoxia-responsive miRNA. miR-210 directly downregulates the expression of iron–sulphur cluster assembly proteins (ISCU) 1 and 2, leading to decreased incorporation of iron–sulphur clusters in proteins involved in mitochondrial metabolism, including Complex I, aconitase (ACO) [76] and SDHB [77] (Fig. 2). In addition, miR-210 has been shown to target and decrease the expression of the CIV assembly protein COX10 [78]. Together, these changes contribute to the reduction in OXPHOS under hypoxia, stimulated by HIF activity.

### *Detoxification and suppression of ROS production*

**Fig. 3:**
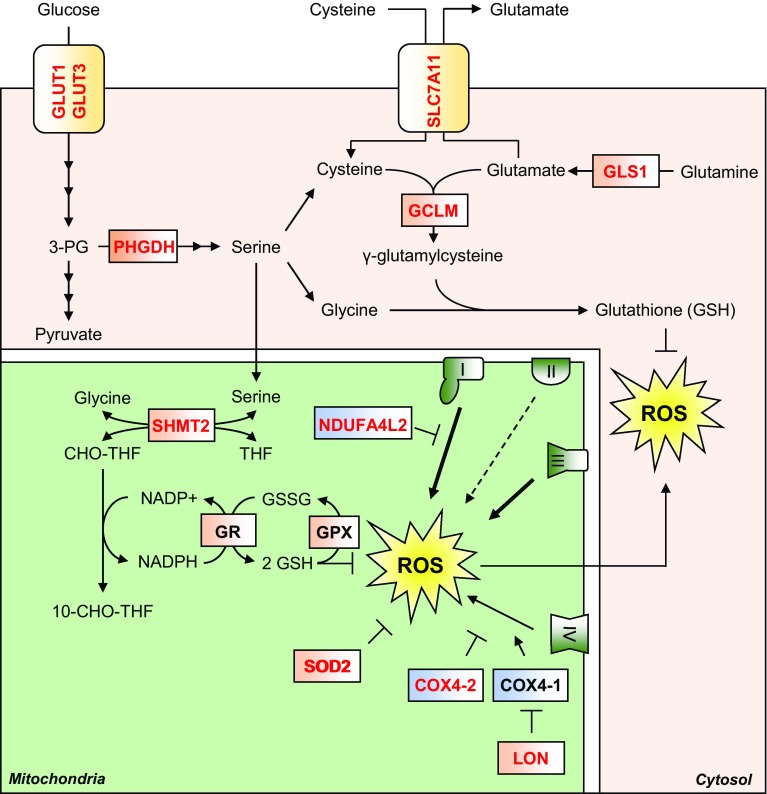
HIF-mediated suppression of ROS. Hypoxia stimulates ROS production, which can damage macromolecules such as proteins, lipids and DNA. HIF signalling upregulates synthesis of the antioxidant tripeptide glutathione by multiple means. HIF upregulates expression of the rate-limiting enzyme in glutathione (GSH) synthesis, GCLM, as well as proteins which increase the cellular levels of the three constituent amino acids of glutathione. Cysteine import is increased by upregulation of SLC7A11, while glutamate synthesis is increased by upregulation of GLS1. Glycine synthesis is increased by increased serine metabolism, first by its synthesis from glycolysis-derived 3-PG, via HIF-dependent upregulation of PHGDH, and second through its conversion to glycine via upregulation of the folate cycle enzyme SHMT2. The folate cycle also produces NADPH, which is utilised by GR to recycle glutathione disulphide (GSSG) to the ROS scavenging GSH. HIF signalling also upregulates the expression of SOD2, a mitochondrial enzyme capable of converting the superoxide free radical to H_2_O_2_. Subunit switching is another way the HIF pathway reduces ROS production is also reduced by HIF-mediated subunit switching of the ETC complexes. The HIFs upregulate expression of an alternative subunit of CI (I), NDUFAL2 which produces less ROS than isoform 1. Similarly, HIFs upregulate an alternative subunit of CIV (IV), COX4-2 which produces less ROS, as well as LON, which degrades isoform 2 (COX4-1)

Hypoxia can stimulate the production of reactive oxygen species (ROS) from the mitochondria, largely from CIII [79], but also from CI and CII [80], as well as from enzymes of the TCA cycle such as OGDH [81]. Mechanistically, decreased CIV activity in hypoxia slows electron transfer along the ETC, increasing the likelihood of unwanted electron transfer to molecular oxygen, which produces the highly reactive superoxide anion (^·^O_2_^−^). ROS can be extremely damaging to cells, causing peroxidation of membrane lipids, redox damage to proteins, and can introduce single-strand breaks into DNA. Because of the potential for ROS to damage the cell, there are several cellular antioxidant defence systems, including detoxifying enzymes, and a large pool of the redox-active tripeptide, glutathione, to absorb free radicals and maintain protein redox states. HIF signalling is responsible for mitigating ROS-mediated damage in hypoxia in a variety of ways (Fig. 3).

Superoxide dismutases (SODs) catalyse the conversion of the superoxide radical to hydrogen peroxide (H_2_O_2_), which in itself is a reactive species, but which can then be converted to harmless water and oxygen by catalase enzymes. In mice, deletion of the *epas1* gene identified that expression of the mitochondrial protein SOD2 was dependent on HIF-2α expression [82], while reporter gene assays in human cells showed that SOD2 expression was induced under hypoxia in a HIF-2 dependent manner [83]. Interestingly, SOD2 expression has also been shown to be suppressed under hypoxia in renal carcinoma cells in a HIF-1 dependent manner, suggesting that SOD2 expression under hypoxia is context-specific [84], and may represent one of the opposing facets of HIF-1 and HIF-2 activity.

The tripeptide glutathione (γ‐l‐glutamyl‐l‐cysteinylglycine) represents another major defence against ROS [85]. Glutathione (represented as GSH) maintains protein redox status by serving as an electron donor, and is capable of reducing and breaking disulphide bonds in proteins that have been oxidised during oxidative stresses such as hypoxia. In the process, disulphide bonds are formed between cysteine thiol groups on adjacent molecules of glutathione, to produce glutathione disulphide (represented as GSSG) [85]. In addition, glutathione can directly detoxify hydrogen peroxide as a substrate of the peroxiredoxin (Prx) and glutathione peroxidase (GPx) enzymes [85, 86]. Glutathione disulphide (GSSG) is recycled to its monomeric form by the reducing power of NADPH, in a reaction catalysed by the enzyme glutathione reductase (GSR) [87]. As the reducing power of NADPH is essential for the recycling of glutathione and maintenance of this antioxidant defence, several key NADPH-producing pathways are upregulated under hypoxic conditions. Serine synthesis is one such pathway that generates NADPH, and HIF-1 has been shown to upregulate several pathway enzymes, such as phosphoglycerate dehydrogenase (PHGDH) [88], and the mitochondrial enzyme serine hydroxymethyltransferase 2 (SHMT2) in a MYC-dependent manner [89].

Not only does HIF-1 activity help to maintain glutathione in its decreased form, it also contributes to increased de novo glutathione synthesis (Fig. 3). HIF-1 is responsible for both upregulating enzymes directly involved in glutathione biosynthesis, and also enzymes involved in the biosynthesis of the three constituent amino acids of glutathione [88–90]. For example, while the serine biosynthetic pathway is an important source of NADPH, serine is also an important precursor for the synthesis of glycine and cysteine [91]. Thus, the HIF-1 dependent upregulation of serine synthesis pathway enzymes in hypoxia increases serine availability for glycine and cysteine synthesis. Furthermore, HIF-1 is responsible for the hypoxic upregulation of solute carrier 7 family member 11 (SLC7A11), which is a component of the xCT cysteine import channel [90], thus increasing cysteine flux into the cell. SLC7A11 is an antiporter which exports one molecule of glutamate for every molecule of cysteine imported, but glutamate is the third component amino acid of glutathione, and so export of glutamate via SLC7A11 would inhibit glutathione synthesis by depleting intracellular glutamate levels. To counteract this, glutamate synthesis from glutamine is increased through HIF-dependent upregulation of the glutaminase 1 and 2 enzymes (GLS1, 2) in the cytosol and mitochondria. Recent work has shown that HIF-1α stabilisation by hypoxia or *EGLN1* (PHD2) deletion in periosteal progenitor cells stimulates GLS1 expression, and thus increases cellular glutathione levels, which protects these cells from ROS-mediated cell death [72]. Furthermore, HIF-1 has been shown to directly stimulate glutathione synthesis in breast cancer cells by upregulating the enzyme responsible for the rate-limiting step in the pathway, namely glutamate–cysteine ligase (GCLM) [90].

Finally, while the HIFs bolster a cell’s antioxidant defence, they are also responsible for minimising ROS production from the mitochondria in hypoxia. This is achieved firstly by reducing mitochondrial mass, as described in detail below, and also through regulating the expression of alternative isoforms of subunits of the respiratory complexes (Fig. 3). The CI subunit NADH dehydrogenase [ubiquinone] 1 alpha sub complex, 4-like 2 (NDUFAL2), is strongly induced in hypoxia in a HIF-1 dependent manner, and acts to decrease ETC activity and mitochondrial ROS production [92]. The mechanism by which NDUFA4L2 decreases CI activity remains unknown, but it is induced in hypoxia in different cell types, and its expression is negatively correlated with expression of all other CI subunits in hypoxia. Similarly, HIF-1 decreases ROS production by upregulating an isoform of the CIV subunit COX4, namely cytochrome *c* oxidase subunit 4 isoform 2 (COX4-2), which makes electron transfer and oxygen consumption more efficient in hypoxia [93]. In parallel, HIF-1 upregulates the mitochondrial lon protease (LON), which is required for the degradation of the less efficient COX4-1 subunit [93].

Together, these studies show that HIF signalling regulates mitochondrial ROS production and detoxification at multiple levels, which is essential for the maintenance of cell viability in hypoxia. It is important to note that there is a reciprocal relationship between HIF signalling and ROS, since ROS are capable of regulating HIF-α stabilisation under hypoxia, which is discussed in more detail.

### *Downregulating mitochondrial mass*

New mitochondria are synthesised in advance of cell division [94], in response to bioenergetic demand [95], and to replace damaged or unwanted mitochondria that have been cleared by mitochondrial autophagy (mitophagy) [96]. While mitochondria possess a small circular genome, mitochondrial biogenesis is largely regulated by the action of a number of nuclear-encoded genes. The first genes implicated in mitochondrial biogenesis were the nuclear respiratory factors (NRFs) 1 and 2, which are transcription factors that regulate the expression of many genes involved in OXPHOS, including all ten nuclear CIV subunit genes [97, 98]. Along with the NRFs, the orphan nuclear receptor estrogen-related receptor alpha (ERRα) [99] and the initiator element binding factor yin yang 1 (YY1) [100] are also involved in the expression of genes involved in mitochondrial function and biogenesis. The transcriptional activity of each of these nuclear factors is dependent on the expression and activity of the PGC family of transcriptional co-activators, which includes PGC-1α, PGC-1β, and PRC. Energetic stress can be signalled via AMP-activated protein kinase (AMPK) and sirtuin 1 (SIRT-1) to activate PGC-1α by phosphorylation [101] and deacetylation [95], respectively. Activation of PGC-1α and its subsequent association with other nuclear factors stimulates the expression of numerous genes involved in mitochondrial biogenesis, including genes that regulate replication such as DNA-directed RNA polymerase mitochondrial (*POLRMT*), as well as transcription and translation, such as transcription factor A mitochondrial (*TFAM*) of the mitochondrial genome [102].

There is considerable overlap between the AMPK and HIF signalling pathways, as both are involved in responding to energetic stresses, though the relationship is complex, with both opposing and co-operative outcomes depending on the context. For example, both AMPK and HIF increase glucose uptake [40, 103], glycolytic flux [54, 104] and autophagy [105, 106], and both suppress protein translation via mTOR [107, 108]. AMPK is also a potent stimulator of mitochondrial biogenesis as described above, which in normoxic conditions acts to restore ATP homeostasis. However, under hypoxia which constitutes an energetic stress, and thus leads to AMPK activation [109], additional mitochondrial biogenesis and oxygen consumption would only exacerbate the stress caused by decreased oxygen availability, and thus HIF signalling under prolonged hypoxia generally acts to decrease mitochondrial mass. Thus, the HIF and AMPK signalling pathways respond to specific but related stresses, and are able to indirectly influence each other depending on the cellular context. A more detailed discussion of the relationship between HIF and AMPK signalling can be found elsewhere [110].

**Fig. 4:**
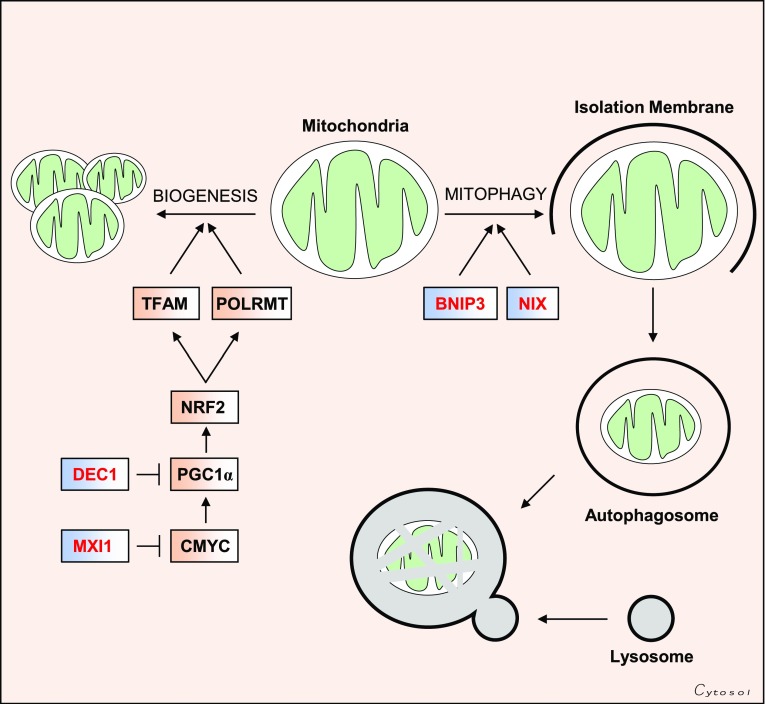
HIF-mediated regulation of mitochondrial mass. The HIF pathway reduces mitochondrial number in the cell by suppressing mitochondrial biogenesis and increasing mitochondrial degradation through mitophagy. Mitochondrial biogenesis is regulated by the PGC-1α pathway, which upregulates mitochondrial proteins required for expression of genes encoded by the mitochondrial genome, such as TFAM and POLRMT. This is achieved through HIF-dependent upregulation of two negative regulators of PGC-1α activity, namely MXI1, which inhibits C-MYC-directed PGC-1α expression, and DEC1, which inhibits PGC-1α transcription by binding to its promoter. HIF signalling also upregulates the expression of two related proteins expressed on the mitochondrial outer membrane, namely BNIP3 and NIX (*BNIP3L*). These proteins flag mitochondria for degradation by the autophagy pathway

The relationship between HIFs and mitochondrial biogenesis has primarily been investigated in renal carcinoma cells, which are commonly deficient in pVHL activity [43, 111, 112]. Loss of pVHL leads to constitutive stabilisation of HIF-α subunits, as well as constitutive expression of HIF-regulated genes. Microarray studies have shown that pVHL deficiency and constitutive HIF activation leads to the upregulation of genes involved the suppression of oxidative phosphorylation, while reconstitution of pVHL reverses these gene changes, and increases both mitochondrial mass, ETC activity and oxygen consumption rates [57, 113]. One of these genes, MAX-interactor 1 (*MXI1*) encodes a negative regulator of C-MYC expression and activity, and thus constitutive MXI1 expression decreases C-MYC-dependent expression of PGC-1α, which suppresses mitochondrial biogenesis [113] (Fig. 4). While it appears that HIF-1 is unequivocally an antagonist of C-MYC, the relationship between C-MYC and HIF-2 is less clear. Co-immunoprecipitation experiments have shown that HIF-1α associates with and sequesters various cofactors required for C-MYC activity, including SP1 and MAX [41]. Conversely, HIF-2α overexpression increases C-MYC binding to these same cofactors, and increases the expression of C-MYC-regulated genes, such as cyclin D1 (*CCND1*) and transcription factor E2F1 (*E2F1*) to promote proliferation [41]. The expectation might then be that HIF-1 and HIF-2 have differential effects on mitochondrial biogenesis. However, a separate study showed that shRNA-mediated silencing of both HIF-1α and HIF-2α in pVHL deficient renal carcinoma cells suppressed MXI1 expression, leading to increased C-MYC-dependent PGC-1α expression, and increased mitochondrial biogenesis [57]. Both HIF-1 and HIF-2 have also been shown to positively regulate the expression of another transcriptional repressor, deleted in esophageal cancer 1 (DEC1), which suppresses PGC-1α expression [57], and leads to decreased mitochondrial biogenesis. Thus, while the regulation of mitochondrial biogenesis may represent one feature of the regulation of metabolism that differs between HIF-1 and HIF-2 in a context-specific manner, taken together the evidence suggests that hypoxia (and HIF) stimulates a reduction in mitochondrial biogenesis.

In addition to the suppression of mitochondrial biogenesis, it has been reported that hypoxia induces mitochondrial turnover [114], through organelle-specific autophagy, termed ‘mitophagy’. Autophagy is a bulk degradative process which leads to the lysosomal digestion of cellular contents, including whole organelles when they are damaged or unwanted. Mitochondria are flagged for autophagy by various means, and each encourages the interaction of the outer mitochondrial membrane with nascent autophagocytic membranes, that eventually surround and isolate the unwanted mitochondrion (Fig. 4). Two such autophagic tags are the closely related proteins BCL2/adenovirus E1B 19 kDa protein-interacting protein 3 (BNIP3) and BCL2/adenovirus E1B 19 kDa protein-interacting protein 3-like (BNIP3L/NIX) [105], both of which are localised to the outer mitochondrial membrane, and are HIF-regulated genes that are strongly induced under hypoxia [115, 116]. While BNIP3 and NIX expression stimulate the expression of biochemical markers of autophagy, such as LC3B lipidation [117], hypoxia-induced mitophagy has yet to be confirmed by more direct methods such as electron microscopy. Thus, while HIF-dependent BNIP3 and NIX expression correlate with decreased mitochondrial mass, the direct relationship between HIF signalling and mitophagy is incompletely understood.

### *Changing mitochondrial distribution*

The name ‘mitochondria’ was coined in 1898 by Carl Benda, and is derived from the Greek words ‘mitos’ meaning ‘thread’, and ‘chondros’ meaning ‘granule’. This describes the dual nature of mitochondria, as an interconnected network of discrete compartments. The mitochondrial network is a highly dynamic cellular compartment, both in terms of its distribution and its morphology. The advent of live-cell imaging has uncovered the degree to which the mitochondrial network behaves like a single organelle that is in constant flux with regard to its continuity and its localisation. In the majority of resting cells, the mitochondrial network is primarily reticulated and distributed throughout the cytoplasm, but mitochondria are far from static, and have varying degrees of motility depending on the cell type and context. In neurons, for example, mitochondria travel greater distances than in other cells because of their axon which can vary in length, and the high energetic demand of the remote synaptic terminal. Defects in mitochondrial trafficking have been identified in a number of neurodegenerative diseases in humans, including Alzheimer’s disease [118] and Huntington’s disease [119], while genetic deletion of various trafficking proteins leads to neuronal phenotypes in mice [120] and drosophila [121]. Together, these examples indicate that the nervous system is particularly reliant on effective mitochondrial trafficking, which is likely due to the functionality of the neuronal type and length of their axons.

**Fig. 5:**
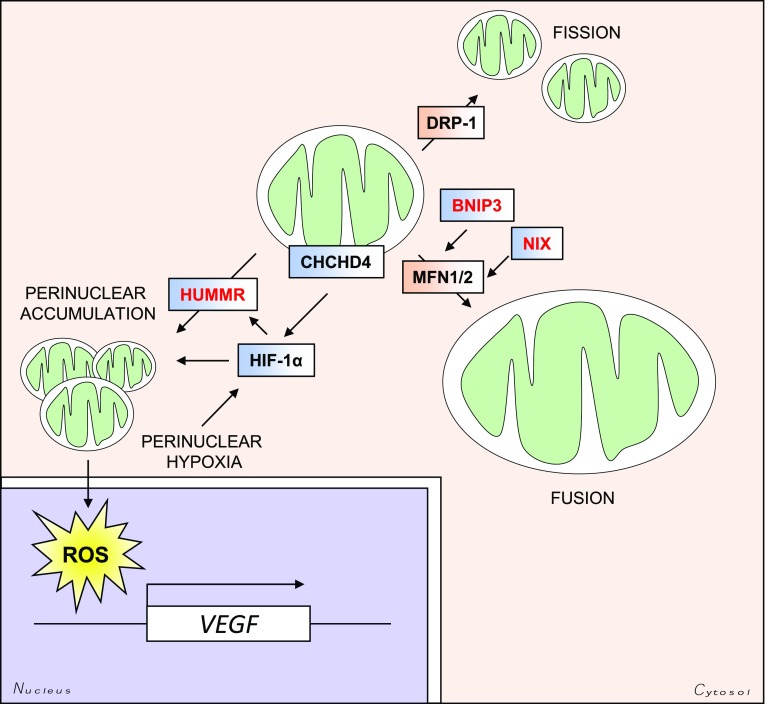
HIF-mediated regulation of mitochondrial size and intracellular distribution. HIF targets regulate the subcellular distribution of mitochondria. The HIF-target HUMMR regulates mitochondrial trafficking along microtubules, and promotes perinuclear clustering of the mitochondrial network. The mitochondrial DRS protein CHCHD4 also stimulates perinuclear clustering of the mitochondria in a HIF-dependent manner. Perinuclear accumulation of the mitochondria stimulates ROS-mediated HIF-dependent upregulation of VEGF transcription. The HIF-regulated genes BNIP3 and NIX (*BNIP3L*) are involved in the MFN1- and MFN2-dependent fusion of mitochondria. Enlargement of the mitochondria confers increased mitochondrial membrane integrity, and protection against apoptosis

Hypoxia has been identified as one of the few bona fide physiological stimuli to induce a shift in the distribution of the mitochondrial network, and in all cases this has been reported as a retrograde redistribution towards the nucleus (Fig. 5). A perinuclear accumulation of the mitochondrial network has been described after short (3 h) and long (72 h) exposures to hypoxia. This shift in distribution is microtubule-dependent, and appears to be required for the efficient delivery of mitochondrial ROS to the nuclei, for the full activation of the promoter regions of certain HIF-1α target genes, including vascular endothelial growth factor (VEGF) [122].

Hypoxia-upregulated mitochondrial movement regulator (HUMMR) is a HIF-1α-upregulated gene that has been identified as a regulator of the interaction between mitochondria and the cytoskeleton via the trafficking proteins mitochondrial Rho GTPase 1 and 2 (MIRO1, 2) [123]. Mitochondrial movement occurs along microtubules (MTs) in a retrograde (towards the MT minus-end) or anterograde (towards the MT plus-end) fashion. The polarity of the microtubules is defined relative to the microtubule organizing centre (MTOC), whose intracellular localization depends on the cell-type and cellular context (e.g. cell-cycle stage) [124]. Hypoxic upregulation of HUMMR leads to both elevated anterograde movement of the mitochondria, and increases mitochondrial axonal content which is dependent on HIF-1α [123]. In addition, exogenous overexpression of HUMMR in astrocytes and the tumour cell line HEK-293 leads to a collapse of the mitochondrial network around the perinucleus [123]. HUMMR may therefore represent a biomechanical link between hypoxia and changes in the intracellular distribution of mitochondria, but is likely to be cell-type- and context-specific. Similarly the mitochondrial IMS protein coiled-coil helix domain containing protein 4 (CHCHD4, Mia40 in yeast) has also been shown to stimulate mitochondrial perinuclear clustering, and thus intracellular oxygenation, in a HIF-1α-dependent manner in U2OS osteosarcoma cells [125]. Perinuclear mitochondrial clustering has also been described in a number of other physiological settings, including fertilization and embryonic development [126]. Since hypoxia is a key stimulant of vasculogenesis during development, and HIF-1α is essential for embryogenesis, it is possible that the relationship between hypoxia signalling and mitochondrial distribution plays an important role during development. Indeed, overexpression of HUMMR (MGARP in mice) in mouse neocortical cells leads to aberrant mitochondrial transport, as well as defects in neocortical development [127].

### *Changing mitochondrial morphology*

In addition to regulation of the subcellular distribution of mitochondria within the mitochondrial network outlined above, changes in mitochondrial morphology are also highly regulated. Mitochondria undergo repeated fission and fusion events, which together is referred to as mitochondrial dynamics. Mitochondrial dynamics are regulated by the opposing functions of a core group of GTPases, as well as a number of accessory and regulatory proteins. Studies of these GTPases have shown that mitochondrial dynamics are also determinants of mitochondrial distribution. Overexpression of the fusion inducing protein mitofusin 2 (MFN2) leads to enlargement of the mitochondria, as well as perinuclear clustering of the mitochondrial network [128]. Similarly, overexpression of a dominant negative form of the fission inducing protein dynamin-related protein (DRP1) encoded by the *DNML1* gene, leads to hyperfusion of the mitochondrial network and perinuclear aggregation [129] (Fig. 5).

Hypoxia and mitochondrial dynamics are closely connected, as has been shown by a number of studies [122, 125, 130, 131]. Under chronic hypoxia (72 h) mitochondria have been observed to enlarge through additional MFN1-dependent (hyper) fusion and decreased DRP1-dependent fission in cancer cells [130]. The HIF-1α target genes *BNIP3* and *BNIP3L* (NIX) also appear to play an important role, and together this elevated mitochondrial fusion confers resistance to apoptosis by improving mitochondrial membrane integrity [130]. Shorter exposures (24 h) to hypoxia in NIH 3T3 cells on the other hand appear to promote mitochondrial fission, through mechanisms which are only partially dependent on HIF-1α. Instead, degradation of the mitochondrial scaffolding protein A-kinase anchor protein 1 (AKAP121), by SIAH2, relieves AKAP121-mediated suppression of DRP1 activity [131]. SIAH2 regulates HIF-1α stability, through the downregulation of PHDs 1 and 3 under hypoxia, and therefore may play a role in regulating the HIF-dependent changes in mitochondrial dynamics and distribution.

Hypoxia-reoxygenation stress has also been shown to result in the appearance of shorter mitochondria due to impaired ATP synthesis [132]. In addition, short exposures to hypoxia (1 h) in glucose-free medium, or hypoxia-reoxygenation in glucose-containing medium leads to the formation of toroidal mitochondria, due to anomalous fusion events caused by swelling and detachment from the cytoskeleton [132]. However, since these effects occur after hypoxia exposures shorter than those generally required for HIF-mediated transcriptional responses, it is likely they are independent of HIF activity. The effect of hypoxia on mitochondrial dynamics appears therefore to be time-dependent, and also dependent on the underlying nutrient availability to cells.

## Mitochondrial regulation of HIF signalling

**Fig. 6:**
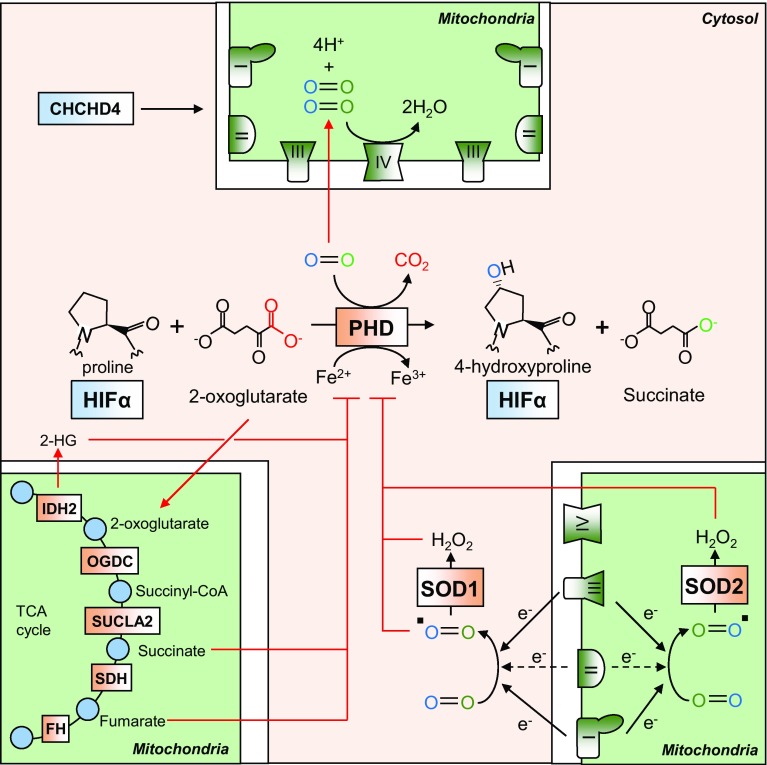
Mitochondrial regulation of HIF signalling. Mitochondrial oxygen consumption at CIV (IV) regulates the intracellular availability of oxygen, which is required for hydroxylation of HIF-α subunits by the PHD enzymes. The mitochondria also metabolise the PHD substrate 2-OG, and consequently regulate its intracellular levels. Two products of mitochondrial 2-OG metabolism, succinate and fumarate, are capable of competitively inhibiting PHD activity, and thus TCA cycle enzyme activity influences intracellular levels of these metabolites, and influence PHD-mediated HIF-α hydroxylation. Disease-associated mutations in IDH2 cause a neoenzymatic reaction which produces 2-HG which is also a competitive inhibitor of the PHD enzymes. ROS production by the mitochondria also regulates PHD activity by influencing the REDOX state of the ferrous (Fe) ion cofactor

As mitochondria are the major oxygen-consuming organelles of the cell, it is perhaps unsurprising that they are capable of influencing the oxygen-dependent degradation of both HIF-1α and HIF-2α subunits [133]. Indeed, ethidium bromide-mediated depletion of mitochondria to generate *ρ*^0^ cells is capable of blocking HIF induction in hypoxia, though the HIF response remains intact under exogenous anoxia ([O_2_] ≤ 0.1%) [79]. In addition, the use of ETC inhibitors, such as rotenone (CI) and antimycin A (CIII), or knockdown of subunits of ETC complexes inhibits the hypoxic stabilisation of HIF-α proteins. The mechanisms by which mitochondria regulate HIF signalling (that have been experimentally demonstrated) all appear to converge on PHD-mediated hydroxylation of the HIF-α subunits (Fig. 6).

### *The availability of molecular oxygen*

Oxygen-dependent hydroxylation is the primary mechanism by which the PHD and FIH enzymes transmit the signal of changes in cellular oxygenation to the HIF machinery. Measurements of intracellular oxygenation using phosphorescence lifetime imaging [134] or immunofluorescence imaging of the nitroimidazole agent pimonidazole in cells [125] have demonstrated that regions of low oxygenation co-localise with the mitochondria [125, 134]. Another imaging study using renilla luciferase to measure intracellular oxygen demonstrated that ETC inhibition redistributed oxygen towards the PHDs [135]. It has also been shown that mitochondrial oxygen consumption at CIV of the ETC influences intracellular oxygenation and hypoxia, as well as HIF-1α stabilisation [125]. Additionally, stimulation of mitochondrial biogenesis through exogenous overexpression of PGC-1α leads to increased HIF signalling through an elevation in mitochondrial OCR and intracellular hypoxia [136]. This is of particular interest in cancer when allied with the observation that PGC-1α upregulation is a critical feature of circulating tumour cells in a mouse xenograft model of breast cancer metastasis, and is strongly correlated with distant metastases in breast cancer patients [137]. Thus, mitochondrial biogenesis is not only experimentally linked to HIF signalling through PGC-1α, but also to tumour cell dissemination. Moreover, there has been repeated demonstration that chemical inhibition of the ETC using a variety of inhibitors of complexes I, III and IV is capable of blocking hypoxic HIF-α induction in hypoxia [138–140]. Together, these studies demonstrate the importance of the mitochondria and their oxygen-consuming activity in regulating the stabilisation of HIF-α proteins and HIF signalling under hypoxia.

### *TCA cycle intermediates*

The hydroxylation of HIF-α by PHDs requires 2-oxoglutarate (2-OG), and produces succinate as a by-product [31, 32]. Both of these metabolites are freely diffusible intermediates of the TCA cycle, which takes place exclusively in the matrix of the mitochondria (Fig. 6). Elevated levels of succinate are capable of inhibiting the hydroxylation reaction [141], as is elevation in the levels of a second TCA cycle intermediate, fumarate [142]. Thus, the intracellular ratio of 2-OG-to-succinate or fumarate greatly influences PHD activity, and the degree of HIF-α stabilisation in both hypoxia and normoxia [142]. Indeed, treatment of succinate dehydrogenase (SDH, CII)-deficient cells with cell-permeable 2-OG derivatives is capable of reversing normoxic HIF-α expression by competitively reversing the inhibition of PHDs caused by succinate accumulation [143]. In addition, pharmacological activators of HIF signalling include 2-OG analogues, such as dimethyloxalylglycine (DMOG) [144] and *N*-oxalylglycine (NOG). In a disease setting, inactivating mutations in the enzyme fumarate hydratase (FH) or subunits of succinate dehydrogenase (SDH) or its assembly factors (SDHAF1, 2) are causative for certain rare tumour syndromes [145–147] in which constitutive HIF stabilisation is detectable and is thought to contribute to disease progression [148]. The primary metabolic defect in SDH and FH deficiency is an accumulation of succinate or the closely related compound fumarate, respectively. Both metabolites are capable of inhibiting PHD and FIH activity by competitive occupation of the enzymatic active site, thus blocking the hydroxylation and subsequent degradation of HIF-α subunits [141, 142]. Despite the constitutive stabilisation of HIF-α in these cases, PHD inhibition and constitutive HIF signalling does not appear to be critical for tumorigenesis, as silencing of HIF-1β does not reverse the increased expression of genes involved in regulating epithelial-to-mesenchymal transition (EMT) [149]. Furthermore, cyst formation was not inhibited in double *Fh1/Hif*-*1α* KO mice, and these cysts also grew larger, suggesting that *HIF1A* may indeed be a tumour suppressor in this context [150]. Instead, the accumulation of these metabolites also inhibits the TET family of oxygen-dependent dioxygenases, responsible for the demethylation and expression of an antimetastatic miRNA cluster. Loss of expression of these miRNAs leads to the expression of an EMT gene signature, including increases in the expression of vimentin, and loss of expression of E-cadherin [149].

Mutations in the TCA enzyme isocitrate dehydrogenase 2 (IDH2), or its cytoplasmic homologue IDH1 are commonly detected in gliomas [151] and certain types of AML [152], as well as in cases of non-malignant metabolic disorders such as d-2-hydroxyglutaric aciduria [153]. The disease-associated active-site mutations cause a neo-enzymatic reaction in which 2-OG is reduced to 2-hydroxyglutarate (2-HG) by NADPH reduction [154]. As a metabolite that is closely related to 2-OG, 2-HG competitively inhibits the PHDs, leading to constitutive HIF-α stabilisation and activity [155]. Indeed, 2-HG has been shown to accumulate under hypoxia via promiscuous metabolism of 2-OG by the malate dehydrogenase (MDH) and lactate dehydrogenase (LDH) enzymes, leading to enhanced hypoxic stabilisation of HIF-α proteins [156]. However, as with fumarate and succinate accumulation, the primary oncogenic influence of 2-HG accumulation appears not to be due to HIF-α stabilisation and activation. Instead, 2-HG is also capable of inhibiting histone demethylases which, like the PHDs, are oxygen-dependent dioxygenases, leading to increases in methylation and the blocking of malignant cell differentiation [157].

### *Reactive oxygen species*

As discussed above, several mitochondrial ETC complexes are a significant source of ROS, and ROS production is elevated under hypoxia. Exogenous treatment of cells with ROS such as H_2_O_2_ is capable of leading to normoxic HIF-1α accumulation in both wild-type HEK293T cells, and HEK293T cells lacking mitochondria [158]. It has also been shown that mitochondrially derived ROS are required for maximal HIF-α protein stabilisation under hypoxia [158], and the ROS-scavenging antioxidant ebselen is capable of blocking HIF-1α stabilisation [79] and its binding to HREs [159]. Using mitochondrially encoded cytochrome *b*-deleted cybrids of HEK293T cells, the primary source of HIF-inducing ROS has been identified as CIII, and that this activity relies on the *Q*_0_ site within the complex [160]. It appears that inhibitors of CIII differentially influence HIF stabilisation and signalling depending on their ability to induce ROS production from CIII [158]. It has been proposed that the mechanism by which ROS lead to HIF stabilisation is due to their effect on the redox state of the ferrous cofactor in the active site of the PHDs [161]. By oxidising this group from a 2 + to a 3 + state, ROS are thought to decrease HIF-α hydroxylation and pVHL recognition [161] (Fig. 6).

The role of ROS in HIF signalling has been reviewed recently [162]. While there is no doubt that mitochondria are significant sources of ROS in both normoxia and hypoxia [79, 163], it still remains unclear as to when endogenously produced mitochondrial ROS are required for HIF signalling. Much of the uncertainty arises from experimental models that do not unequivocally distinguish between the effects of ROS production and oxygen consumption. Interestingly, in a study by Chua et al. [138] they proposed that the ROS-producing activity of CIII was not required for HIF-1α protein stabilisation in 143B cells. Hypoxic HIF-1α protein stabilisation was similarly blocked by inhibitors of CI, CIII and CIV, and furthermore, inhibition of CIII activity using either a ROS-inducing inhibitor of CIII (antimycin A) or a ROS-reducing inhibitor of CIII (myxothiazol) both blocked hypoxic HIF-1α stabilisation [138]. In addition, myxothiazol completely inhibited oxygen consumption (at CIV), while co-treatment with TMPD which donates electrons to CIV via cytochrome *c*, was capable of restoring both oxygen consumption and HIF-1α stabilisation [138]. This same study demonstrated that exogenous H_2_O_2_ had no direct influence on PHD activity in vitro [138], calling into question the mechanistic basis for ROS-mediated HIF-1α stabilisation. Finally, several studies have shown that exogenous ROS scavengers such as N-acetylcysteine (NAC) and MnTBAP are incapable of influencing hypoxic stabilisation of HIF-1α under conditions of CIII inhibition [135], hypoxia [138], or elevated oxygen consumption through CHCHD4 overexpression [139]. Thus further mechanistic work to clarify the connection between ROS and HIF-1α protein stabilisation and the precise molecular mechanisms involved is needed.

## Closing remarks

As oxygen is central to mitochondrial metabolism it is not surprising that the HIF-mediated response to hypoxia involves a global cellular response allowing cells to metabolically adapt and survive when oxygen is limiting. Hypoxia-mediated metabolic adaptations involve changes in the regulatory control of key molecular components of metabolic pathways involving mitochondria, as well as dynamic changes in the morphology, mass and subcellular localization of mitochondria themselves. Since mitochondria are of such fundamental importance to oxygen-dependent metabolism, it is perhaps also not surprising that HIF-mediated adaptations to hypoxia impinge on mitochondrial function at many levels. In general it is clear that the aim of HIF-mediated adaptation to hypoxia is to decrease mitochondrial activity, and thus a cell’s reliance on oxygen for survival. Intriguingly, HIF-1α protein has been detected in mitochondrial fractions, suggesting the possibility that HIF-1α protein has a direct, non-transcriptional effect on mitochondria [164].

What is perhaps less well appreciated is the reciprocal nature of the relationship between HIF signalling and mitochondria. The HIFs are responsive to perturbations of mitochondrial biochemistry, and are thus in many ways sensors of mitochondrial health. Conversely, the mitochondria are capable of transmitting numerous metabolic stresses to the HIF pathway, and thus participate centrally in HIF signalling. Hypoxia is a fundamental feature of metazoan life, and underlies physiological processes during development as well as pathophysiological processes involved in diseases such as cancer. The relationship between hypoxia signalling and mitochondria is important in diverse biological contexts, and therefore warrants continued investigation and expansion of our understanding.
